# Inward Rectifier K^+^ Currents Are Regulated by CaMKII in Endothelial Cells of Primarily Cultured Bovine Pulmonary Arteries

**DOI:** 10.1371/journal.pone.0145508

**Published:** 2015-12-23

**Authors:** Lihui Qu, Lei Yu, Yanli Wang, Xin Jin, Qianlong Zhang, Ping Lu, Xiufeng Yu, Weiwei Zhong, Xiaodong Zheng, Ningren Cui, Chun Jiang, Daling Zhu

**Affiliations:** 1 Department of Physiology, College of Basic Medical Sciences, Harbin Medical University-Daqing, Daqing 163319, Heilongjiang, China; 2 Department of Biopharmaceutical Sciences, College of Pharmacy, Harbin Medical University-Daqing, Daqing 163319, Heilongjiang, China; 3 Biopharmaceutical Key Laboratory of Heilongjiang Province, Harbin 150086, Heilongjiang, China; 4 Department of Biology, Georgia State University, 50 Decatur Street, Atlanta, Georgia, 30302, United States of America; 5 Department of Rheumatology and Immunology, First Affiliated Hospital of Harbin Medical University, Harbin 150086, Heilongjiang, China; Indiana University School of Medicine, UNITED STATES

## Abstract

Endothelium lines the interior surface of vascular walls and regulates vascular tones. The endothelial cells sense and respond to chemical and mechanical stimuli in the circulation, and couple the stimulus signals to vascular smooth muscles, in which inward rectifier K^+^ currents (Kir) play an important role. Here we applied several complementary strategies to determine the Kir subunit in primarily cultured pulmonary arterial endothelial cells (PAECs) that was regulated by the Ca^2+^/calmodulin (CaM)-dependent protein kinase II (CaMKII). In whole-cell voltage clamp, the Kir currents were sensitive to micromolar concentrations of extracellular Ba^2+^. In excised inside-out patches, an inward rectifier K^+^ current was observed with single-channel conductance 32.43 ± 0.45 pS and P_open_ 0.27 ± 0.04, which were consistent with known unitary conductance of Kir 2.1. RT-PCR and western blot results showed that expression of Kir 2.1 was significantly stronger than that of other subtypes in PAECs. Pharmacological analysis of the Kir currents demonstrated that insensitivity to intracellular ATP, pinacidil, glibenclamide, pH, GDP-β-S and choleratoxin suggested that currents weren’t determined by K_ATP_, Kir2.3, Kir2.4 and Kir3.x. The currents were strongly suppressed by exposure to CaMKII inhibitor W-7 and KN-62. The expression of Kir2.1 was inhibited by knocking down CaMKII. Consistently, vasodilation was suppressed by Ba^2+^, W-7 and KN-62 in isolated and perfused pulmonary arterial rings. These results suggest that the PAECs express an inward rectifier K^+^ current that is carried dominantly by Kir2.1, and this K^+^ channel appears to be targeted by CaMKII-dependent intracellular signaling systems.

## Introduction

The single layer of endothelium in the vasculature is the interface between circulating blood and vessel walls, where a number of vascular regulations take place. Responding to circulating hormones, metabolites and other chemical and mechanical stimuli, the endothelial cells (ECs) release several vasoactive substances, such as nitric oxide (NO), endothelins, prostaglandins, prostacyclin and thromboxane A2, etc. that then act on smooth muscle cells (SMCs) affecting the vascular tension. Another SMC-regulating substance is K^+^ which is known as an endothelium-derived hyperpolarizing factor. These K^+^ ions are released into the interstitial space by SMCs as a result of muscle contractions, and then up-taken by ECs [1]. A rapid clearance of these extracellular K^+^ ions prevents excessive SMC depolarization and sustained smooth muscle contraction. This important regulatory mechanism relies on the membrane K^+^ channels and transport. However, the K^+^-transporting channels are not well understood in ECs with respect to their molecular identity and regulation.

One of the most important EC regulations is carried out by Ca^2+^/calmodulin-dependent protein kinase II (CaMKII) which is activated by shear stress and oxidative stress [2]. Several known down-stream targets of CaMKII include the endothelial nitric oxide synthase (eNOS) [3,4], actin cytoskeletons [2,5] and voltage-gated K^+^ channel [2]. Another potential target of CaMKII is the inward rectifier K^+^ channels (Kir) known to be expressed in ECs. These K^+^ channels are thought to be important in maintaining EC resting membrane potentials [6]. It is also possible that the modulation of the Kir channels affects the interstitial K^+^ concentrations as well as vascular smooth muscle contractility, as some members in the Kir channel family play a role in K^+^ transport in astrocytes [7], retinal Müller cells [8], and renal tubular epithelium [9]. Indeed, the shear stress causes vasorelaxation by producing hyperpolarization of aortic ECs via activation of Kir channels [10–12].

In this study, we found evidence for the regulation of a Kir channel by CaMKII in primarily cultured PAECs from bovine pulmonary arteries. The biophysical properties, functional evidence and molecular identity suggest that this Kir channel is carried dominantly by Kir2.1.

## Materials and Methods

### Ethics statement

Calf lungs from local slaughterhouse were used to harvest ECs. All animal experiments were performed in compliance with an approved protocol by the Ethical Committee of Laboratory Animals at Harbin Medical University.

### Endothelial cell culture

Primary cultured PAECs were prepared from pulmonary arteries isolated from calf lungs as described previously [13]. The arteries were cut open along their length with the inner surface exposed under sterile condition. The inner surface was then gently scratched with a surgical blade to obtain ECs. The Harvested ECs were then transferred to media Dulbecco's modified eagle's medium (DMEM) containing 20% fetal bovine serum (FBS) and 1mg/ml collagenase for 15min. Digested ECs were then centrifuged for 5min at 2000 rpm. Supernatant fluid was discarded, and ECs were washed three times with PBS and then cultured with DMEM containing 20% FBS. The purity and identity of PAECs were confirmed to be above 90% with positive immunofluorescence staining using antibodies to CD31 (Santa Cruz).

### Patch-clamp recordings

Patch-clamp experiments were performed at room temperature. Patch pipettes were pulled from borosilicate glass capillaries by a pipette puller (Sutter P-97) and firepolished by a microforge (MF830, Narashige, Japan). The tip resistance was 2 to 4 MΩ. Ionic currents were recorded with a patch amplifier (Axon 200B, Molecular Devices). Single-channel currents were recorded from outside out and cell-attached configurations. Current records were low-pass filtered (2 kHz, Bessel 4-pole filter, -3 dB), digitized (20 kHz, 16-bit resolution), and stored on a computer hard drive for later analysis using Clampfit 10.0 software (Molecular Devices). The extracellular solution contained (in mM): 10 KCl, 135 potassium gluconate, 5 EGTA, 5 glucose, and 10 HEPES (pH = 7.4). In physical condition, 135 NaCl was used instead of potassium gluconate. The intracellular solution contained (in mM): 10 KCl, 133 potassium gluconate, 5 EGTA, 5 glucose, 1 K_2_ATP, 0.5 NaADP, and 10 HEPES (pH = 7.4).

Single-channel conductance was measured with slope command potentials from 100 to -100 mV. The open-state probability (P_open_) was calculated by first measuring the time, *tj*, spent at current levels corresponding to *j* = 0, 1, 2, … N channels open, based on all evident openings during the entire period of record [14]. The P_open_ was then obtained as P_open_ = (ΣN j _= 1_t_j_j)/TN, where N was the number of channels active in the patch, and T was the duration of recordings. Dwell times were studied using Fetchex 6.0 software (Molecular Devices) in a duration of 40–70 s. Open and closed times were measured from records in which only a single active channel was observed. The open- and closed-time distributions were fitted using the Marquardt-LSQ method in the Pstat6 software (Axon Instruments Inc.). Open/closed events shorter than 0.2 ms were ignored, as a result of the use of 1000 Hz offline filter [14].

### Reverse transcription PCR

Total RNA was extracted from primarily cultured PAECs using the Trizol reagent (Invitrogen) following the manufacturer’s instructions. The concentration of RNA was measured at 260 nm with ultraviolet spectrophotometry. The extracted total RNA was reverse-transcribed to cDNA using the Superscript first-strand cDNA synthesis kit (Invitrogen). PCR was performed with the cDNA to amplify desired fragment in a DNA thermal cycler (Thermo Scientific, Waltham, MD, USA). Gene-specific primers were listed in Table 1. The PCR products were separated by electrophoresis on 2% agarose gel and stained with ethidium bromide. β-actin was used as an internal control.

**Table 1:** Gene-specific primers designed and expected length of RT-PCR products.

Kir Gene		Primer Sequence	Size, bp
Kir1.1	Forward	5¢- TTATTCTTCATCTCCCCACTGA -3¢	349
	Reverse	5¢- GGTTGTCGTAGCCTCTTTTCAT-3¢	
Kir2.1	Forward	5¢-GTTGGTGGGGTGTTTGACTT -3¢	328
	Reverse	5¢- ACGAGAGGGAGAGAGAGAATCA -3¢	
Kir2.2	Forward	5¢- GGCAACCTACGCAAGAGC-3¢	236
	Reverse	5¢-TCGAAGTCATCCGTTTCCAG-3¢	
Kir2.4	Forward	5¢-GCCACTTCCATCGCACTTAT-3¢	184
	Reverse	5¢-GCCTCCTCTTCCTCATCTTCTT-3¢	
Kir4.1	Forward	5¢- CCACCAAAACCTATGGAGAGAA -3¢	308
	Reverse	5¢- ACAGGGAAACAGGGCTACCT -3¢	
Kir5.1	Forward	5¢- CCTGGGAAGTAGACAAGGAAGA -3¢	435
	Reverse	5¢- GGATGTAGGACAGCGAGAAGAT -3¢	
Kir6.1	Forward	5¢- TTTTACCTCTGCTTTCCTCTTCTC -3¢	332
	Reverse	5¢- CTGGGGTCGTGGTTTTCTT -3¢	
Kir6.2	Forward	5¢- TAGGAAGGGCAGGTGATGAG -3¢	364
	Reverse	5¢- AGCAATCCAAAGGCTCTCTAAG -3¢	
β-actin	Forward	5¢-TCCGTGACATCAAGGAGAAGC-3¢	270
	Reverse	5¢- GCACCGTGTTGGCGTAGAG-3¢	

### siRNA transfection

To knockdown CaMKII expression in cultured PAECs, PAECs were transfected with small interfering RNA (accession numbers NM_001075938.1). The siCaMKII sequence is 5’- GCCUGUACCAGCAGAUCAATT-3’ and non-targeted control siRNA (siNC) sequence is 5’-UUCUCCGAACGUGUCACGUTT-3’, both were designed and synthesized by Gene Pharma. siNC was used to exam and optimize the efficiency of transfection and served as a negative control. siRNA was transfected into PAECs using X-tremeGene siRNA transfection reagent (Roche Applied, Mannheim, Germany) following the manufacturer’s protocol. In brief, 2 μg siRNA and 10 μl X-tremeGene siRNA transfection reagent were incubated with serum-free medium for 5 min respectively, and mixed together. After 20 min incubation at room temperature, the mixture was added to cells. The cells were cultured for 6–8 h, and then washed and subsequently cultured in DMEM containing 5% FBS for 24 h. The efficiency of silencing was detected by western blot.

### Western blot analysis

The cultured PAECs were lysed with ice-cold lysis buffer (Tris 50Mm, pH 7.4, NaCl 150 mM, Triton X-100 1%, glycerol 10%, Nonidet P-40 1%, EDTA 5 mM, and PMSF 2mM) and centrifuged at 13,500 r.p.m for 15 min at 4°C. The supernatant was collected and total protein was determined using BCA protein assay kit with bovine serum albumin as the standard (Beyotime Institute of Biotechnology, China). Samples with equal amount of protein (50 μg) were separated in 10%-12% SDS-PAGE and then transferred onto nitrocellulose membranes (Millipore, MA). The membranes were then blocked with 5% fat-free milk in Tris-buffered saline and 1% Tween-20 (TBS-T) for 1 h and then incubated with the corresponding antibodies overnight at 4°C. After washing with TBS-T, the membranes were incubated with horseradish peroxidase-conjugated goat anti-rabbit antibody or goat anti-mouse antibody, for 2h at room temperature. Immunoreactive bands were visualized by enhanced chemiluminescence (ECL) kit (Pierce, CA) and exposed on an X-ray film. The immunoblots intensities were quantified using Quantity One software (BioRad). All antibodies were purchased from Santa Cruz except Kir1.1, Kir2.2, p-CaMKII and CaMKII from Abcam.

### Pulmonary artery (PA) preparation and tension measurement

Rats were deeply anesthetized with pentobarbital sodium (50mg/kg). Lungs were dissected out from thoracic cavities and immersed into cold oxygenated Krebs solution (in mM): NaCl 116, KCl 4.2, CaCl_2_ 2.5, NaH_2_PO_4_ 1.6, MgSO_4_ 1.2, NaHCO_3_ 22, and D-glucose 11, pH 7.4. The endothelium-free rings were prepared by rubbing with a sanded polyethylene tubing and confirmed with acetylcholine. The rings were mounted on a force transducer (ALC-MPA, Shanghai Alcbio Biology Technology Co, Ltd, China) for measurements of isometric force contraction in a water-jacketed organ bath with 2 ml oxygenated Krebs solution bubbled continuously with 95% O_2_ and 5% CO_2_ and maintained at 37°C. All rings were pretested with 1μM phenylephrine (PE) to ensure the tissue vitality. When endothelium was removed, the rings were tested by PE for contraction followed by an exposure to ACh (1 μM). The rings were considered to be endothelium-free if more than 90% relaxation was eliminated. PE and Ach then were washed out and the rings were allowed to equilibrate in the Krebs solution for 30–60 min before experiments. Both basal tension of 0.3g (passive force) and stable ring tension induced by PE were regarded as 100%.

### Chemicals and drugs

W-7, KN-62, GDP-β-S, Cholera toxin, pinacidil, glibenclamide, carbachol, L-NAME and MgATP were purchased from Sigma. Chemicals were prepared in high-concentration stock solution in double-distilled H_2_O or DMSO and were diluted in bath solution to experimental concentrations immediately before usage. In cases where DMSO was used, its concentration was controlled at 0.1% (vol/vol), which did not change the activity of Kir2.x channel. Chemicals were applied to the bath solution.

### Data analysis

Data are presented as means SE. Differences in means were tested with ANOVA and Student’s t-test, and were accepted as significant if *P* ≤ 0.05.

## Result

### Whole-cell K^+^ currents in cultured PAECs

**Fig 1:**
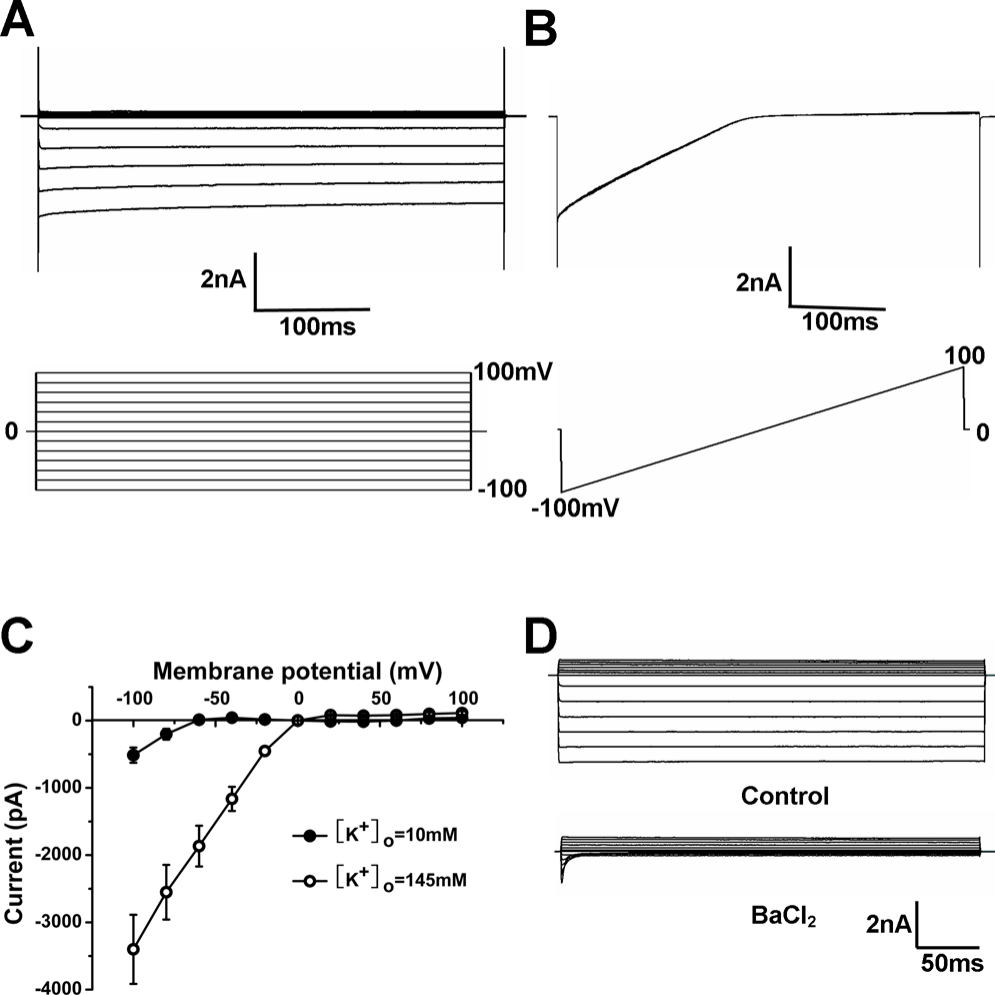
Whole-cell K^+^ currents in cultured PAECs. (A) Representative whole-cell K^+^ currents were recorded in cultured PAECs. The whole cell currents were recorded with a holding potential at 0mV and command pulses from -100 mV to 100 mV. The recording was obtained under symmetrical conditions with bath and pipette solution containing 145mM K^+^. (B) Current traces obtained with a voltage-ramp command from -100mV to 100 mV and the reverse potential was near to 0 mV under symmetrical condition with bath and pipette solutions indicated in (A). (C) Current-voltage relationships were obtained from symmetrical condition indicated in (A) and replacing bath solution for 10 mM K^+^. The inwardly rectifying component was obviously reduced if the extracellular K^+^ concentration is decreased from 145 to 10 mM, and the reversal potential was shifted to –66 ± 1 mV. (D) Exposure of the cells to Ba^2+^ led to a strong inhibition of these K^+^ currents in the inward direction with very little effect on the outward currents.

PAECs in passages 3–5 were chosen for patch clamp. The cells were transferred to a cover slide one day before the experiment. The cells on the cover slide were transferred to a recording chamber and allowed to settle for a few minutes before patch. Whole-cell K^+^ currents were recorded in voltage-clamp with symmetric concentrations of K^+^ (145 mM) applied to the bath and pipette solutions so that the reversal potential is near to 0 mV. The membrane potential was stepped to command pulses from -100 mV to 100 mV. Under this condition, the PAECs displayed large currents (Fig 1A). The currents were showed with a ramp command from -100 mV to 100 mV (Fig 1B). The *I-V* relationship indicated that these currents had strong inward rectification with large and linear conductance in negative membrane potentials, much smaller conductance in positive voltages (Fig 1C). The inwardly rectifier component was obviously reduced if the extracellular K^+^ concentration was decreased from 145 to 10 mM, and the reversal potential was shifted to –66 ± 1 mV, close to the theoretical reversal potential of a K^+^-specific current (–68.5 mV), which suggested that the currents were carried by K^+^ (Fig 1C). Exposure of the cells to 100 μM Ba^2+^ led to a strong inhibition of these K^+^ currents in the inward direction with very little effect on the outward currents (Fig 1D). Quantitatively, these Ba^2+^ sensitive currents made up to 90% of the whole-cell currents, suggesting that the inward rectifier K^+^ currents were the predominant K^+^ channels in PAECs.

### Single-channel biophysical properties

**Fig 2:**
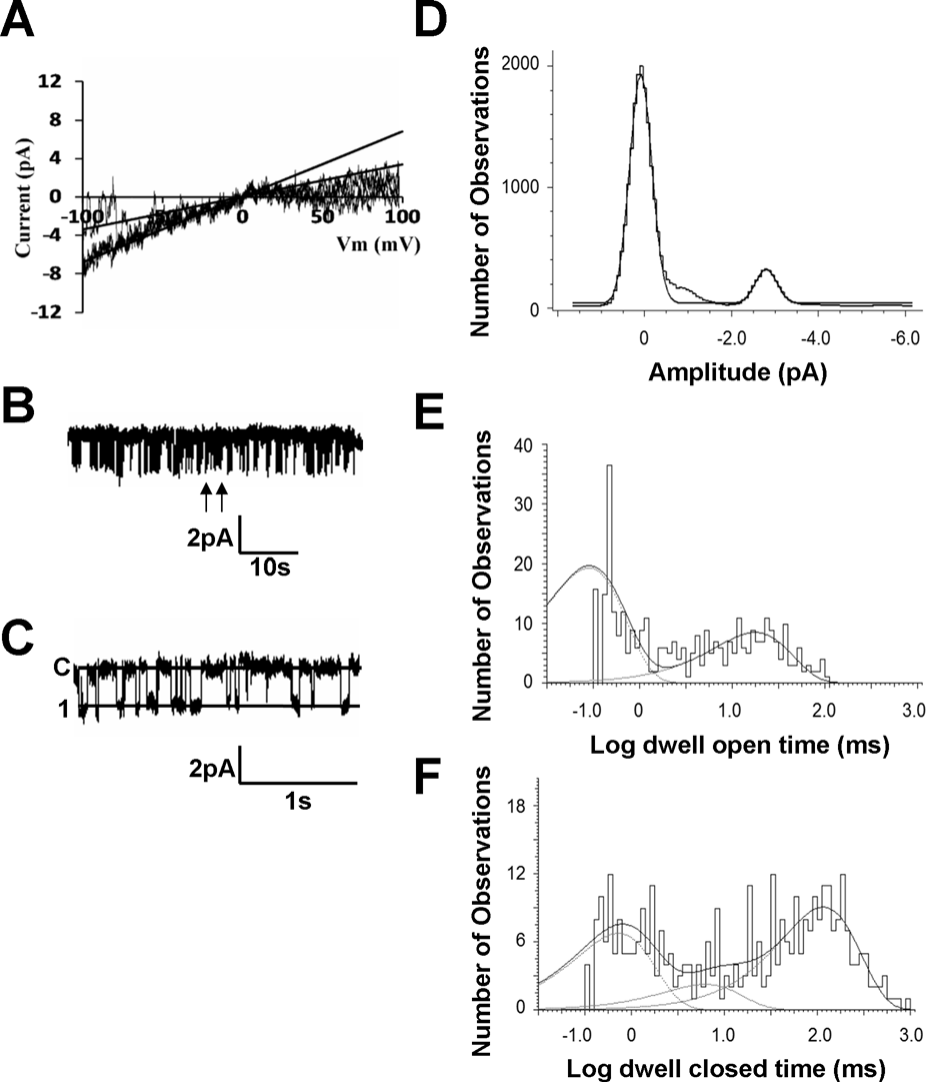
Single-channel biophysical properties of the inward rectifier K^+^ currents. (A) In excised inside-out patches, the currents were recorded under symmetrical conditions with bath and pipette solution containing 145 mM K^+^. (B) Single-channel currents were recorded in a cell attached patch under symmetrical condition indicated in Fig 1. (C) Trace is an expansion from the record of Fig B between arrows. (D) All-point histogram shows a single channel recorded in a patch membrane obtained from a PAEC with amplitude 2.7pA at the holding potential of -80mV. (E) The dwell-time histogram of channel openings was described by two exponential with the constants: τ_O1_ and τ_O2_ are 0.3 ms and 17.8 ms, respectively. (F) The dwell-time histogram for channel closures contained three components of time constants: τ_C1_, τ_C2_ and τ_C3_ are 0.7 ms, 6.2 ms and 115.9 ms, respectively.

To show the biophysical properties of the inward rectifier K^+^ currents, single-channel recordings were performed in excised inside-out and cell-attached patches in the PAECs with equal concentrations of K^+^ on either side of the plasma membranes. In excised inside-out patches, the prominent current had a slope conductance of 32.43 ± 0.5 pS (n = 7) and another 16 ± 0.4 pS (n = 7) in negative membrane potentials (Fig 2A). In cell-attached patches, the open state probability (P_open_) of the 32 pS currents was 0.27 ± 0.04 (n = 5) at -80 mV (Fig 2B and 2C).

Single-channel unitary amplitude was shown in Fig 2D. All-point histogram showed a single channel recorded in a patch membrane obtained from a PAEC with amplitude 2.7 pA at the holding potential of -80mV (Fig 2D). Our results showed that dwell-time histograms of channel openings (n = 4 patches) were described with two exponentials with time constant τ_O1_ 0.5 ± 0.1 ms and τ_O2_ 14.2 ± 1.8 ms (Fig 2E). The dwell-time histograms for closure of the Kir channel were described with three exponentials (τ_C1_ 0.6 ± 0.1 ms, τ_C2_ 6.2 ± 1.4 ms and τ_C3_ 116.2 ± 10.1 ms) (Fig 2F).

### Molecular identity

**Fig 3:**
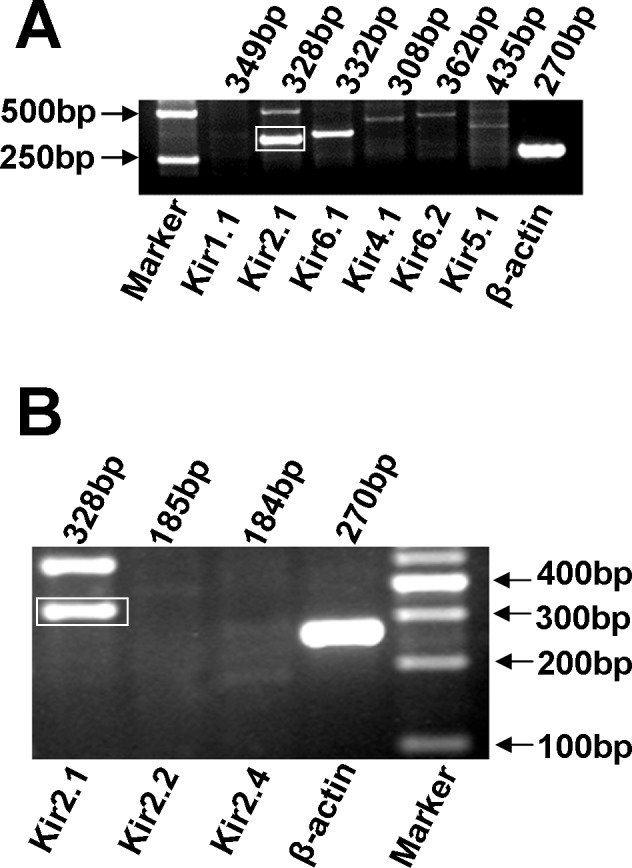
Analysis of mRNA encoding different types of Kir channel subunits. (A) RT-PCR results showed that expression of Kir2.1 was significantly stronger than those of other subtypes (n = 3). (B) The expression of Kir2.1 was more clearer compared with the other Kir 2.x subtypes.

To elucidate the molecular identity of these Kir currents, mRNAs were obtained from the PAECs. RT-PCR analysis was carried out to detect expressions of Kir1.1, Kir 2.1, Kir2.2, Kir2.4, Kir 4.1, Kir5.1, Kir6.1 and Kir6.2 in PAECs. The results showed that the expression of Kir 2.1 was significantly stronger than that of other subtypes (n = 3) (Fig 3).

**Fig 4:**
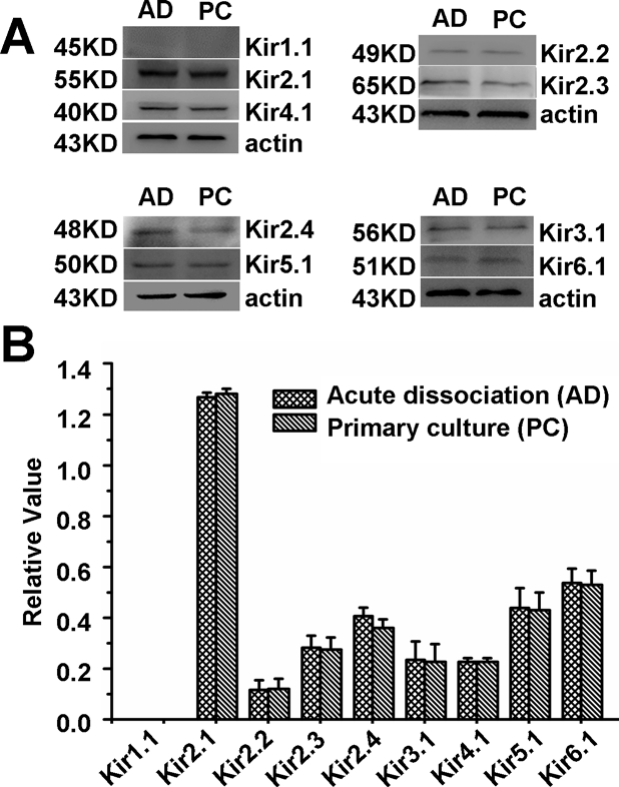
Western blot data analyses for Kir channels. (A) Kirs protein was detected in PAECs of acute dissociation (AD) and primary culture (PC). (B) Expression of Kir 2.1 was significantly more prominent than other subtypes of Kir channels and there were no significant difference of Kirs expression between AD and PC (P < 0.05).

Western blot results for Kir 1.1, Kir 2.1, Kir2.2, Kir2.3, Kir2.4, Kir3.1, Kir 4.1, Kir5.1 and Kir6.1 showed that the Kir2.1 protein was obviously strongerthan that of other subtypes (Fig 4).

### Pharmacological identity

**Fig 5:**
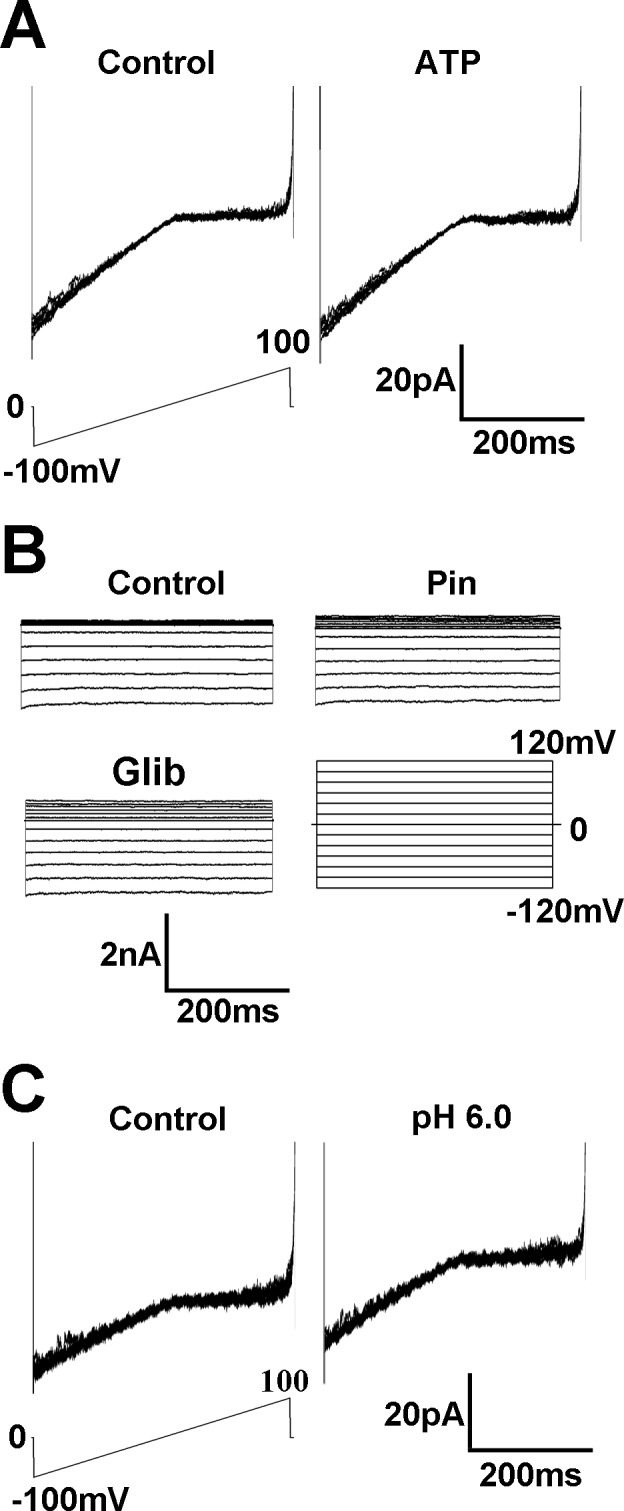
These currents are insensitive to ATP and pH. **(**A) In the inside-out patches, exposure of K_2_ATP (1 mM) on the internal membrane did not have any effect on the currents. (B) Specific K_ATP_ channel opener pinacidil (10 μM) or blocker glibenclamide (10 μM) did not have effect on the inward currents either. (C) In the inside-out patches, pH 6.0 did not produce any detectable inhibition of these K^+^ currents.

Since Kir6.1 was found in our RT-PCR and western blot analysis, ATP-sensitive K^+^ channels were investigated. Inside-out patches, exposure of the internal membrane to ATP (1 mM) did not have any effect on the currents (Fig 5A). These Kir currents were insensitive to the K_ATP_ channel opener pinacidil (10 μM), neither to K_ATP_ channel blocker glibenclamide (10 μM) (Fig 5B), suggesting that these currents were not carried by the K_ATP_ channels.

In the Kir2 channel subfamily, a good number of members such as Kir2.3 and Kir2.4 are pH sensitive. Therefore, we studied the pH sensitivity of the K^+^ currents in inside-out patches. Exposure of the internal membranes to a solution with pH 6.0 did not produce any detectable inhibition of these K^+^ currents (Fig 5C). These results suggested that the Kir currents were neither Kir2.3 nor Kir2.4 currents that showed strong sigmoidal dependence on pH, with the currents being inhibited at low extracellular pH [15] although there were weak expression of Kir2.3 and Kir2.4 according to PCR and western blot tests.

**Fig 6:**
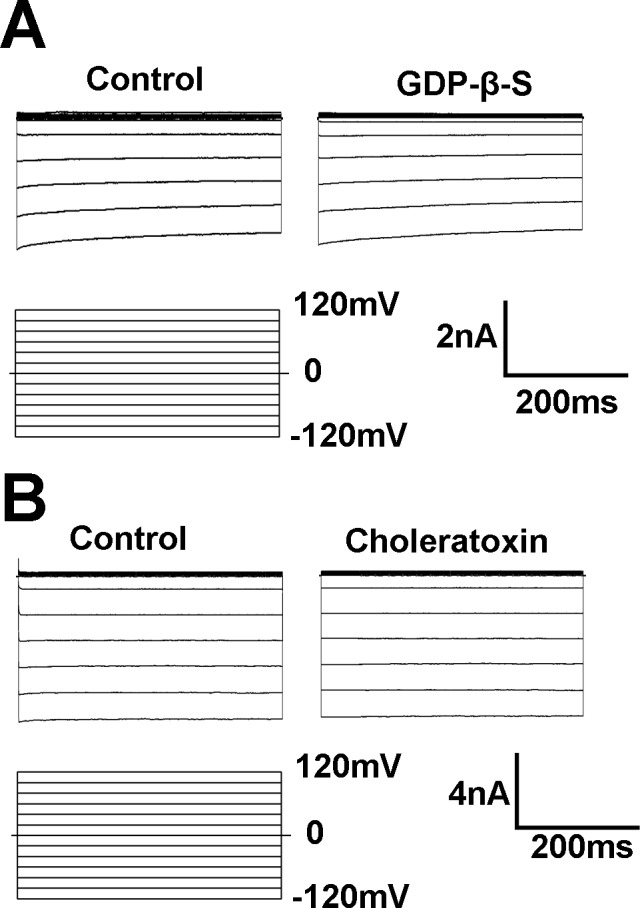
These currents are insensitive to GDP-β-S and choleratoxin. (A) The inhibition of G-protein signaling with the GDP-β-S did not affect the Kir currents. GDP-β-S (1 mM), a non-hydrolysable GTP analog, was applied in pipette internal solution, and whole-cell currents were studied after 15 min. (B) G-protein activator choleratoxin (0.01 M) had no significant effect on the currents in whole-cell recording.

Kir3.x members are G-protein coupled (GIRK) and sensitive to G-protein signaling. If these Kir currents in PAECs are GIRK channels, they should be G-protein sensitive. To test this hypothesis, GDP-β-S (1 mM), a non-hydrolysable GTP analog, was applied in pipette internal solution, and whole-cell currents were recorded after 15 min. The inhibition of G-protein signaling with the GDP-β-S did not affect the Kir currents (Fig 6A). Similarly, activation of G-protein signaling by a treatment of the cells with choleratoxin (0.01μM) had no significant effect on the currents in whole-cell recording (Fig 6B). These results suggested that the Kir currents were not mediated by GIRK channels.

### Modulation by extracellular messengers and protein phosphorylation

**Fig 7:**
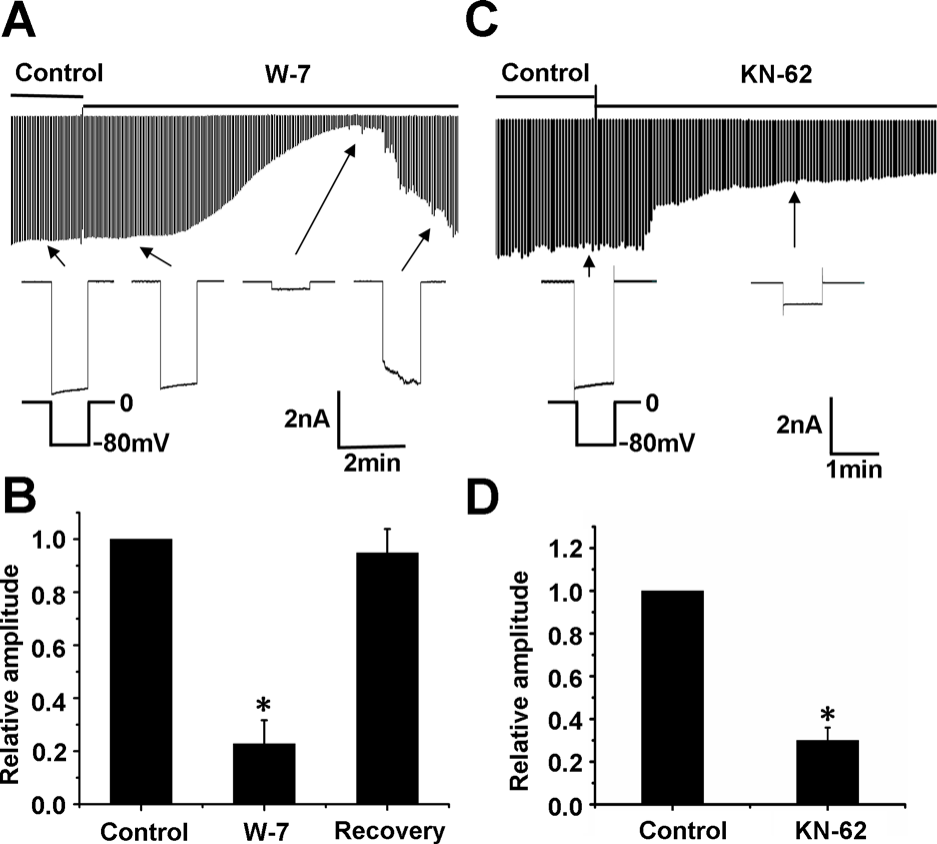
The effects of CaMKII on these currents. (A) W-7 (100 μM) strongly suppressed the Kir currents. The channel inhibition took place rapidly within 1 min after the exposure, lasted for a short period (1–2 min), and then became desensitized. (B) Quantitatively, W-7 inhibited the currents by 77.2 ± 0.1% (n = 9). The channel inhibition was recovered by 94.84% in 5.7 ± 2.1 min after application of W-7 (n = 9). (C) CaMKII inhibitor KN-62 (10 μM) suppressed the Kir currents after PAECs were exposed for 1.5 min and the inhibition was not recovered. (D) Quantitatively, KN-62 inhibited the currents by 71 ± 0.1% (n = 5). ^*^ *P*<0.05 versus control.

Exposure to the CaM inhibitor W-7 (100 μM) strongly suppressed the Kir currents (Fig 7A). Channel inhibition took place rapidly within 1 min after the exposure, lasted for a short period (1–2 min), and then became desensitized. Quantitatively, W-7 inhibited the currents by 77.2 ± 0.1% (n = 9). The channel inhibition was recovered by 94.8% in 5.7 ± 2.1 min (n = 9) after application of W-7 (Fig 7B). Application of the CaMKII inhibitor KN-62 (10 μM) eliminated the currents by 71.0 ± 0.1% (n = 5), but the inhibition was not recovered (Fig 7C and 7D).

**Fig 8:**
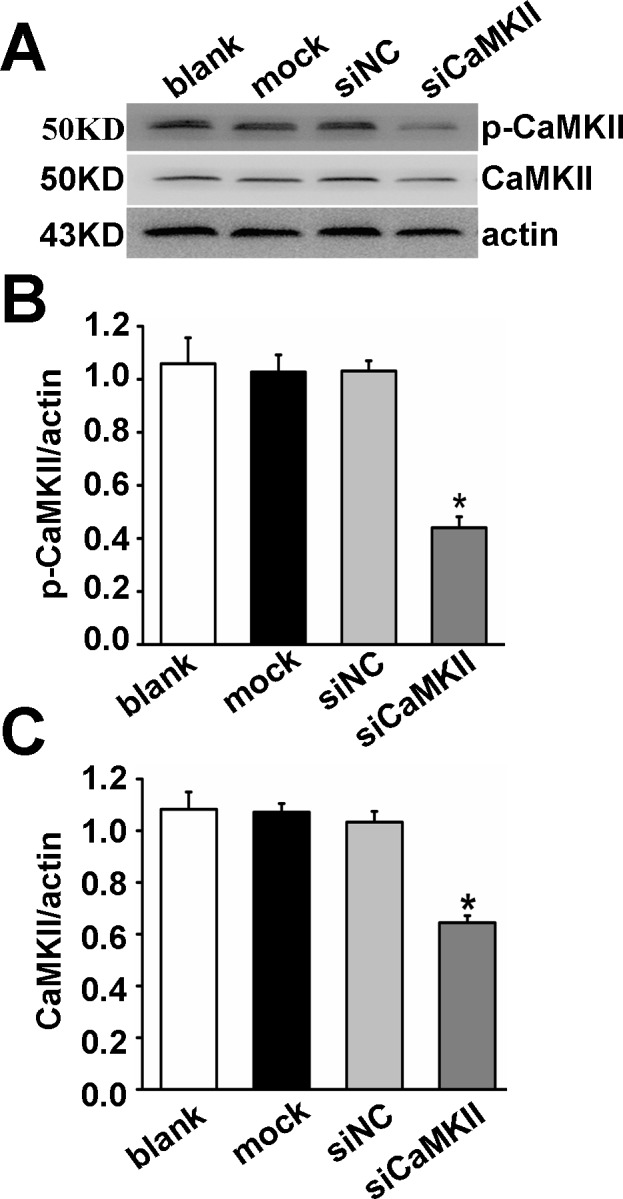
CaMKII was knocked down by siRNA transfection. (A) Western blots analyzed CaMKII expression in untreated PAECs (Blank), PAECs treated with transfection vehicle alone (Mock), non-targeted small interfering RNA control (siNC), and siRNA targeted to CaMKII (siCaMKII). (B, C) p-CaMKII and CaMKII expressions of cells treated with siCaMKII were significantly inhibited (P < 0.05, n = 5). ^*^ *P*<0.05 versus siNC.

**Fig 9:**
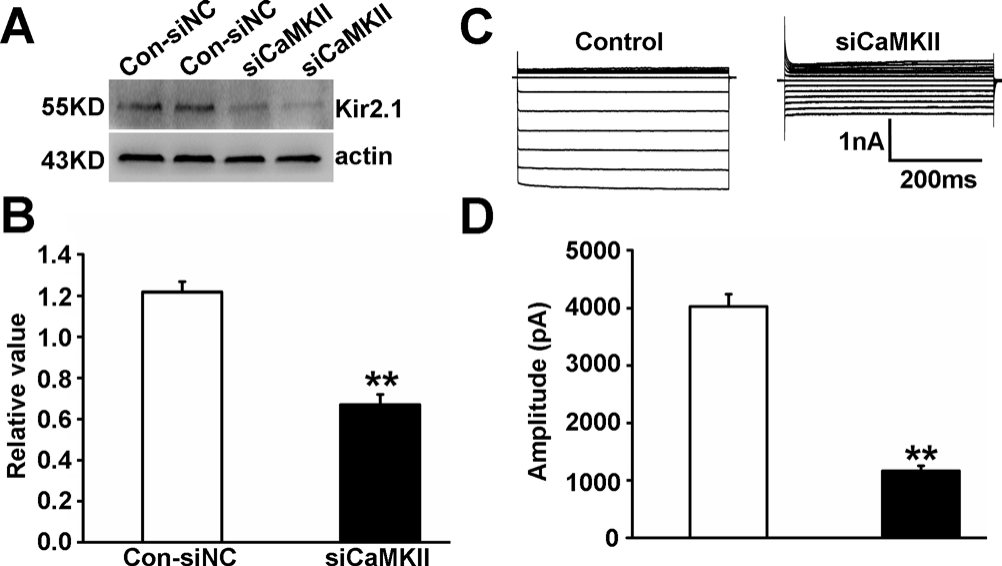
Effect of siCaMKII on Kir 2.1 expression and currents in PAECs. SiCaMKII treatment remarkably reduced Kir 2.1 protein expression (A and B) and currents (C and D) of PEACs compared with siNC (n = 5, P < 0.01).

To further test whether CaMKII was involved in regulation of Kir 2.1 in PAECs, we applied RNA interference technology to knock down CaMKII. The efficiency of the siRNA was measured with western blot analysis for CaMKII protein. Our results showed that there were no differences between cells treated with siNC and untreated cells (Blank) or cells treated only with the transfection reagent (Mock). In contrast, p-CaMKII and CaMKII expressions of cells treated with CaMKII small interfering RNA (siCaMKII) were significantly inhibited compared with siNC (P < 0.05, n = 5, Fig 8). SiCaMKII treatment remarkably reduced Kir 2.1 protein production (Fig 9A and 9B) and currents (Fig 9C and 9D) of PEACs compared with siNC (P < 0.01, n = 5), suggesting that CaMKII was involved in modulation of Kir 2.1.

**Fig 10:**
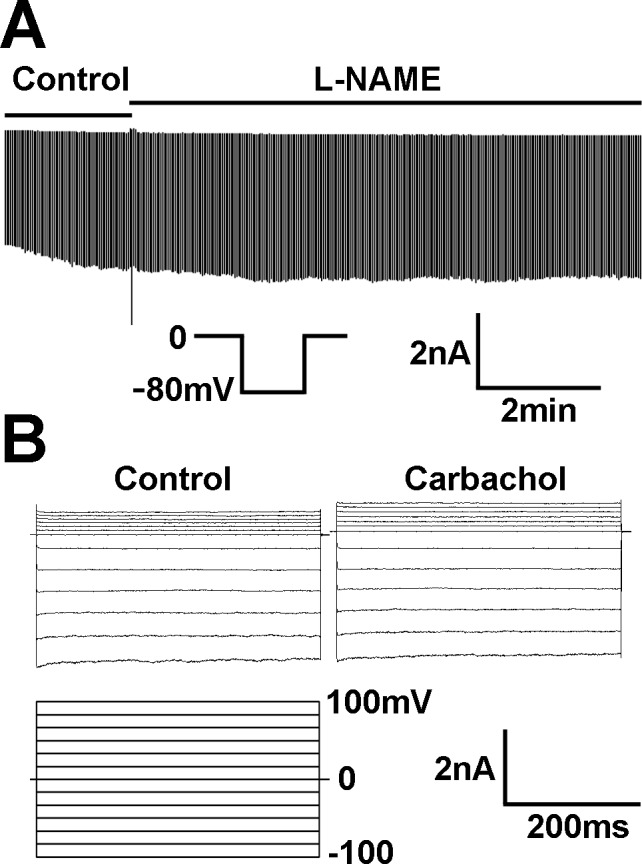
The effects of carbachol and L-NAME on these currents. (A) The Kir currents in PAECs were insensitive to the M receptor agonist carbachol (1 μM). (B) eNOS inhibitor L-NAME (10 μM) had no effect on the current activation.

The Kir currents in PAECs were insensitive to the M receptor agonist carbachol (1 μM), neither to the eNOS inhibitor L-NAME (10 μM) (Fig 10).

### Roles of Kir and CaMKII in the tension of pulmonary artery rings

**Fig 11:**
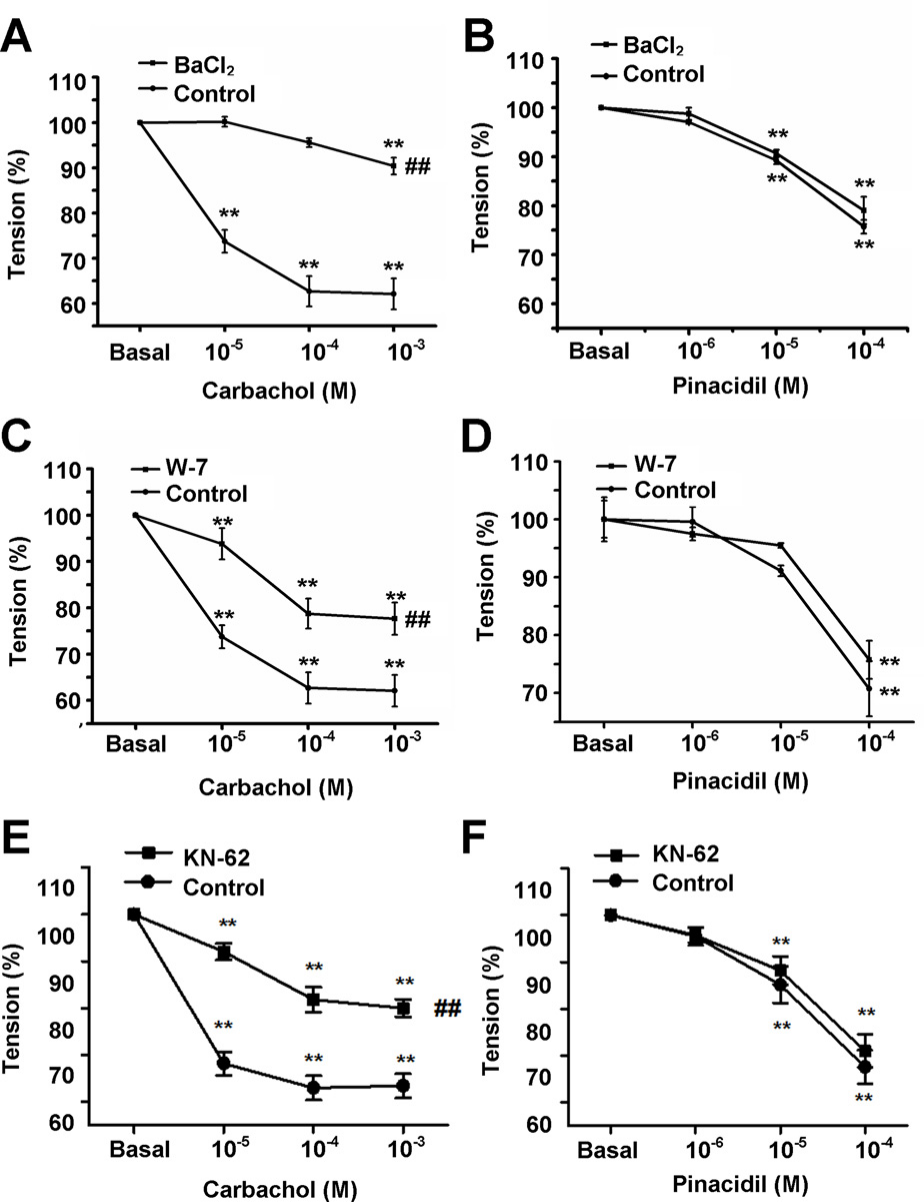
The inhibitive effects of BaCl_2_, W-7 and KN-62on relaxation of PAs. Pretreated the isolated perfused pulmonary artery rings with BaCl_2_ (100 μM) for 20 min (A), W-7 (100 μM) for 60 min (C) and KN-62 (10 μM)for 20 min (E), the amplitude of relaxation induced by carbachol (1μM) was inhibited significantly (n = 6). When the PA endothelium was removed, the amplitude of relaxation induced by pinacidil was not affected by BaCl_2_ (B), W-7 (D) andKN-62 (F) (n = 6). ^**^ *P*<0.01 versus basal value, ^##^ *P*<0.01 versus control.

In isolated and perfused pulmonary artery (PA) rings, the tension of the artery rings was studied in vitro with force-electricity transducers. In PA endothelium-intact rings, carbachol (1 μM) elicited relaxation after the PA rings were pre-contracted by phenylephrine (PE, 1 μM). To examine the roles of Kir and CaMKII in the tension of PA rings, PA rings were pretreated with Ba^2+^ (100 μM) for 20 min (Fig 11A), W-7 (100 μM) for 60 min (Fig 11C) and KN-62 (10 μM) for 20 min (Fig 11E), respectively and the amplitude of relaxation induced by carbachol was inhibited significantly. When the PA endothelium was removed, the amplitude of relaxation induced by pinacidil was not affected by pretreatment PA rings with Ba^2+^ (Fig 11B), W-7 (Fig 11D) and KN-62 (Fig 11F), respectively. These results suggested that the inhibitive effects of BaCl_2_, W-7 and KN-62on relaxation of PAs were endothelium dependent.

## Discussion

K^+^ conductance is a major determinant of membrane potential in VSMCs and ECs. K^+^ channels in ECs indirectly participate in the control of vascular tones by several mechanisms, e.g., release of nitric oxide and endothelium-derived hyperpolarizing factor [16]. Kir channels composed of Kir subunits are one of the most prominent types of K^+^ channels in ECs, conduct inward currents at potentials more negative than the K^+^ equilibrium potential but permit much smaller currents at potentials positive to the reversal potential and are important players in the control of the resting potential in EC [17]. Our results showed that the currents are sensitive Ba^2+^ and almost completely blocked by 100 μM Ba^2+^, which is in line with findings of other research group on Kir [18]. Although Ba^2+^ is not a selective blocher of Kir, this very concentration of 100 μM Ba^2+^ is relatively selective for Kir, because an effective block of other potassium currents such as Ca^2+^-activated K^+^ currents require a higher dose of Ba^2+^ [19]. The Kir channels are known to express heterogeneously in different EC types [6]. In our study, we have found that PAECs displayed large currents with strong inward rectification. The currents have a reversal potential close to the equilibrium potential of K^+^. In excised inside-out patches, an inward rectifier K^+^ current was identified. The current has strong inward rectification with unitary conductance ~32 pS, which is consistent with a previous study showing a similar current which is considered as Kir 2.1 in PAECs [6,20]. RT-PCR and western blot analysis have shown that the expression level of Kir 2.1 was much higher than those of other subtype of Kir channels. The current is insensitive to changes for pH, so that it is unlikely to belong to the Kir2.3 and Kir 2.4 but similar to the cloned Kir2.1 [21]. Because Kir6 subfamily (K_ATP_) is regulated by intracellular ATP, we exposed the internal membrane to 1 mM ATP, which did not produce any effect on the currents. These Kir currents were insensitive to the K_ATP_ channel opener pinacidil, neither to K_ATP_ channel blocker glibenclamide, suggesting that these currents are not carried by the K_ATP_ channels. The Kir3 subfamily is a G protein-activated strong inwardly rectifier K^+^ channel [22], but the currents were not influenced by the inhibition and activation of G-protein. Together, these results suggest that the PAECs express an inward rectifier K^+^ current that is likely to be Kir2.1.

Membrane potential of aortic endothelial cells under resting conditions is dominated by endogenous endothelial inwardly rectifier K^+^ channels belonging to a family of strong rectifiers and are homologous to Kir2.x in bovine aortic endothelial cells [23]. Kir2.2 and Kir2.1 are primary determinants of endogenous K^+^ conductance in human aortic endothelial cells (HAECs) under resting conditions and that Kir2.2 provides the dominant conductance in these cells [24]. PAECs are normally exposed to conditions different from systemic arteries, such as low blood pressures, high oxygen levels and high pH. These differences may result in expressions of different K^+^ channels in the PAECs serving for different functional needs.

In this study, the Kir currents in PAECs were insensitive to the eNOS inhibitor L-NAME, although some Kir channels are upstream of eNOS [25]. We found that the exposure to the calmodulin inhibitor W-7 and CaMKII inhibitor KN-62 strongly suppresses the K^+^ current. The channel inhibition was transient and returned to the basal level in a few minutes without washout of W-7, but inhibition of KN-62 persisted after washout. Auto-phosphorylation of threonine 286 occurs upon an initial increase in Ca^2+^ and Ca^2+^/CaM binding, resulting in structural changes of the regulatory domain of the CaMKII [2]. W-7 is inhibitor of CaM, but does not inhibit auto-phosphorylation of CaMKII, while KN-62 is relatively specific inhibitor of CaMKII, which is probably the reason that Kir current inhibition by KN-62 was not recovered with washout. In order to confirm our conclusions obtained with CaMKII inhibitors, we applied RNA interference technology to knock down CaMKII and found Kir 2.1 protein expression and currents were inhibited in siCaMKII PAECs. This result provided further evidence for involvement of CaMKII in regulation of Kir 2.1 in PAECs. Consistently, pre-incubation of PAs with W-7 and KN-62 inhibits the relaxation induced by carbachol in endothelium intact PA rings after PA rings are pre-contracted by PE. When the endothelium is removed, the inhibitive effect of W-7 on relaxation induced by pinacidil disappears. The results suggest that the inhibitive effects of W-7 on relaxation induced by carbachol or pinacidil are endothelium-dependent. Thus it is likely that the downstream target of CaMKIIis Kir2.1.

As a major regulator of cell resting membrane potential, K^+^ channel activity is an important determinant of vascular tone and vessel diameter. It is known that inhibition of functional expressions of inward rectifier K^+^ channels contribute to the more depolarized resting membrane potential. In addition, K^+^ is thought to be an endothelium-derived hyperpolarizing factor (EDHF) and plays an important role in the relaxation of artery [1]. Accumulating of K^+^ in the interstitial space may activate Kir2.1 [26] that may transport interstitial K^+^ into PAECs to decrease extracellular K^+^ concentration, and accelerate SMC relaxation. Also Kir in PAECs may be involved in endothelial cell functions, such as intracellular Ca^2+^ signals as well as the production and release of many vasoactive factors, e.g., nitric oxide and PGI_2_. Therefore, the large inward rectifier K^+^ currents are likely to play an important role in the regulation of vascular tones. In this regard, our current studies showing intracellular signal pathway for the Kir channel regulation constitute a significant step toward the understanding of endothelium regulation by K^+^ channel.
